# “To brain or not to brain”: evaluating the possible direct effects of the satiety factor oleoylethanolamide in the central nervous system

**DOI:** 10.3389/fendo.2023.1158287

**Published:** 2023-05-10

**Authors:** Adele Romano, Marzia Friuli, Barbara Eramo, Cristina Anna Gallelli, Justyna Barbara Koczwara, Elnaz Karimian Azari, Adrien Paquot, Myrtha Arnold, Wolfgang Langhans, Giulio G. Muccioli, Thomas Alexander Lutz, Silvana Gaetani

**Affiliations:** ^1^ Department of Physiology and Pharmacology “V. Erspamer”, Sapienza University of Rome, Rome, Italy; ^2^ Physiology and Behavior Laboratory, ETH Zurich, Zurich, Switzerland; ^3^ Bioanalysis and Pharmacology of Bioactive Lipids Research Group, Louvain Drug Research Institute, Université Catholique de Louvain, UCLouvain, Brussels, Belgium; ^4^ Institute of Veterinary Physiology, Vetsuisse Faculty, University of Zurich, Zurich, Switzerland

**Keywords:** brain distribution, hormones, gut-brain axis, N-acylethanolamines, N-oleoylethanolamine, eating behavior

## Abstract

**Introduction:**

Oleoylethanolamide (OEA), an endogenous N-acylethanolamine acting as a gut-to-brain signal to control food intake and metabolism, has been attracting attention as a target for novel therapies against obesity and eating disorders. Numerous observations suggested that the OEA effects might be peripherally mediated, although they involve central pathways including noradrenergic, histaminergic and oxytocinergic systems of the brainstem and the hypothalamus. Whether these pathways are activated directly by OEA or whether they are downstream of afferent nerves is still highly debated. Some early studies suggested vagal afferent fibers as the main route, but our previous observations have contradicted this idea and led us to consider the blood circulation as an alternative way for OEA’s central actions.

**Methods:**

To test this hypothesis, we first investigated the impact of subdiaphragmatic vagal deafferentation (SDA) on the OEA-induced activation of selected brain nuclei. Then, we analyzed the pattern of OEA distribution in plasma and brain at different time points after intraperitoneal administration in addition to measuring food intake.

**Results:**

Confirming and extending our previous findings that subdiaphragmatic vagal afferents are not necessary for the eating-inhibitory effect of exogenous OEA, our present results demonstrate that vagal sensory fibers are also not necessary for the neurochemical effects of OEA. Rather, within a few minutes after intraperitoneal administration, we found an increased concentration of intact OEA in different brain areas, associated with the inhibition of food intake.

**Conclusion:**

Our results support that systemic OEA rapidly reaches the brain *via* the circulation and inhibits eating by acting directly on selected brain nuclei.

## 1 Introduction

Oleoylethanolamide (OEA) is a monounsaturated lipid mediator belonging to the N-acyethanolamine family (NAEs) together with anandamide (AEA), palmitoylethanolamide (PEA), stearoyletanolamide (SEA), linoleoylethanolamide (LEA), and other analogues. OEA is also known as a member of the para-cannabinoid system because of the structural similarity with one of the best characterized endocannabinoids, namely AEA. However, contrary to AEA, OEA does not activate cannabinoid receptors and does not produce cannabino-mimetic effects (1).

Three different receptors have been identified to be activated by OEA, including the peroxisome proliferator-activated receptor alpha (PPAR-α, EC50 of ~120 nM), the G protein-coupled receptor-119 (EC50 ~3 μM), and the transient receptor potential cation channel vanilloid-1 (EC50 of ∼2 μM) (2, 3).

Diet-derived oleic acid stimulates OEA synthesis in the first part of the small intestine of a variety of species including rats and mice (2, 4–6). OEA is metabolized into oleic acid and ethanolamine by the fatty acid amide hydrolase expressed by several tissues, and by the N-acylethanolamine hydrolyzing acid amidase notably expressed in the intestinal epithelium (2, 4, 6).

A plethora of biological functions and pharmacological effects in different domains have been associated with OEA, ranging from neuroprotection to anti-inflammatory actions (7–9), from amelioration of mood disorders (9–11) to the control of satiety and regulation of lipid metabolism (4, 12–20). Most of the studies conducted on OEA over the last two decades have focused on its potential contribution to the development of improved treatments for obesity and eating disorders (4, 12–18) because OEA reduces food intake and body weight gain in obese rodents and humans (4, 21).

Moreover, the anti-obesity effect of OEA is also supported by its capability to control lipid metabolism by reducing serum lipid levels, hepatic lipid accumulation and adipose FAT/CD36 lipid transport in obese rodents (20–22). Finally, we recently provided evidence that OEA exerts a selective inhibitory effect on binge-like eating behavior in female rats (18), thus opening novel perspectives for its potential use for the treatment of eating disorders.

The behavioral effects of exogenous OEA are accompanied by the induction of c-Fos in specific brain areas involved in the control of food intake, such as the area postrema (AP) and the nucleus of the solitary tract (NST) in the brainstem, as well as the tuberomammillary (TMN), paraventricular (PVN), and supraoptic nuclei in the hypothalamus (23). In these areas, OEA activates oxytocinergic, noradrenergic and histaminergic pathways, which seem to play a necessary role in mediating its eating-inhibitory effects (23). In fact, OEA’s effects can be prevented by the central administration of the oxytocin (OXY) receptor antagonist L-368,899 (24), or by the ablation of noradrenergic pathways from the NST to the PVN (13), and they are absent in mice lacking the enzyme histidine-decarboxylase that is crucial for histamine synthesis (14).

Despite the large scientific literature on the pharmacological effects of OEA after exogenous administration (7, 8, 25–34), the route by which an OEA-induced signal is conveyed from the periphery to the brain is still debated. This question is very relevant because OEA has received increasing attention worldwide for its pharmacological properties, and further elucidation of its mechanism of action might help to clarify the pharmacodynamic and bioavailability properties, two crucial aspects for planning clinical trials aimed at evaluating novel OEA-based treatments.

Some previous observations had suggested the involvement of visceral vagal afferents (4, 35), because the OEA-induced inhibition of eating was prevented in rats that underwent either a total subdiaphragmatic vagotomy or in rats treated with a neurotoxic dose of capsaicin (4, 35). However, as we previously pointed out (15), these two procedures have limits. On one hand, total subdiaphragmatic vagotomy abolishes both vagal afferent and efferent fibers, impairing the physiological bi-directional crosstalk between gut and brain (36). On the other hand, capsaicin treatment is not specific for vagal afferents (37), as it causes neurotoxic damage of unmyelinated visceral sensory neurons of both vagal and spinal afferents. Furthermore, it has been demonstrated that capsaicin exerts neurotoxic effects also on neurons of the AP and NST, which receive projections from unmyelinated primary sensory neurons (38, 39).

Finally, in our previous collaborative study (15), we selectively investigated the role of vagal afferents in the behavioral effects of OEA by subjecting rats to subdiaphragmatic vagal deafferentation (SDA), a surgery that eliminates all abdominal vagal afferents while sparing approximately half of the efferents (40). Our findings demonstrated that vagal afferents from below the diaphragm are not necessary for the behavioral effects of intraperitoneally (i.p.) injected OEA, which inhibited eating similarly in both SDA lesioned and SHAM-operated control rats (15). This result led us to hypothesize an alternative pathway by which OEA might act on the brain. In keeping with such a hypothesis, more recently we also demonstrated that the surgical ablation of the AP completely prevented both the behavioral and the neurochemical effects of OEA (17). This finding supports a role of the AP, a circumventricular organ devoid of a functional blood brain barrier, as a putative receptive brain region for circulating OEA, leading us to consider the blood circulation as the primary way for OEA to reach the brain after systemic administration.

To test this hypothesis, now we built on our previous observation that SDA does not prevent OEA’s behavioral effects and examined whether vagal afferents are necessary for the central neurochemical effects of the drug. Therefore, as a first step (Experiment 1) in the present study, we performed an immunohistochemical analysis of the pattern of c-Fos and dopamine beta-hydroxylase (DBH) expressions in hypothalamic and brainstem areas of rats that underwent SDA or SHAM surgery (15) and that were i.p. injected with OEA at the dose (10 mg kg^-1^) previously demonstrated to inhibit food intake.

As a second step (Experiment 2), we investigated in another set of rats whether the dynamics of the OEA distribution in plasma and in selected brain areas at different time points after its i.p. administration corresponded to the timelines of the food intake inhibition. For this purpose, we measured food intake until the pertinent points of sacrifice and assessed by UPLC-MS/MS the levels of OEA in the plasma and selected brain areas collected from control and OEA-treated rats at 2.5, 5, 15, 30, or 60 min after acute administration (10 mg kg^-1^, i.p.). The concentrations of other NAEs and 2-arachidonoylglycerol (2-AG) were also measured in the brain and in the blood plasma.

## 2 Materials and methods

### 2.1 Drugs and treatments (for both experiments 1 and 2)

Following our previous studies (16–18), 10 mg kg^-1^ OEA in a vehicle (VEH) solution (2 ml kg^-1^) of saline/polyethylene glycol/Tween 80 (90/5/5, v/v/v) was administered by i.p. injection. Control animals received a bolus of VEH (2 ml kg^-1^). Both VEH and OEA solutions were freshly prepared on each test day and administered at dark onset. OEA was purchased from Sigma Aldrich (St. Louis, Missouri, USA).

### 2.2 Experiment 1

#### 2.2.1 Role of ascending subdiaphragmatic vagal fibers in OEA-induced activation of selected brain nuclei

##### 2.2.1.1 Animals and housing

To investigate the role of ascending subdiaphragmatic vagal fibers in OEA-induced activation of selected brain nuclei, in Experiment 1, we analyzed brains collected from rats that had been subjected to SDA surgery and behaviorally tested in a previous study (15), in which we evaluated the role of vagal afferents in the behavioral effects of systemically administered OEA. The detailed protocol regarding animal housing, catheter implantation and SDA functional test was reported in our previous study (15). Briefly, 27 male Sprague Dawley rats (Charles Rivers, Sulzfeld, Germany) were individually housed and subjected to SDA (n=15) or SHAM (n=12) surgery. Additionally, during the same surgery, rats were equipped with i.p. catheters for OEA or VEH administration. SDA surgery was functionally verified as previously described, by the absence of cholecystokinin (CCK)-induced satiation (41, 42). Specifically, after 4-h food deprivation, 4 μg/kg of CCK-8 (Bachem, Budendorf, Switzerland) or saline were injected *via* the i.p. catheter and 30-min food intake was measured. In SHAM rats, CCK-8 reduced 30-min food intake 52.1 ± 6% (means ± SE) compared with saline. On the basis of previous studies (41), a maximum of 30% food intake reduction by CCK was set as a threshold for complete SDA surgeries. All SDA animals met this criterion and were included, together with SHAM rats, in the behavioral test that allowed us to demonstrate that OEA pro-satiety effects were evident in both SHAM and SDA rats (as reported in (15). After a 3-day wash-out period from the behavioral test all rats were maintained in a free-feeding condition and were treated again with either OEA (10 mg kg^-1^, i.p.) or VEH at dark onset. 2 hours after treatment they were deeply anesthetized with pentobarbital sodium (80 mg kg^-1^; Kantonsapotheke, Zurich, Switzerland), and transcardially perfused with ice-cold phosphate buffered saline (PBS), followed by 4% paraformaldehyde (PFA) fixative solution. Fixed brains were removed from the skull, collected, postfixed overnight, cryoprotected in 20% sucrose-phosphate buffer, and then snap frozen in dry-ice-cold 2-methylbutane (-60°C), to be stored at -80°C until processed in the Experiment 1 of the present study. All experiments were performed upon the approval of the Veterinary Office of the Canton of Zurich and according to the European Community directives 2010/63/EU.

##### 2.2.1.2 Immunohistochemical analyses

**Figure 1:**
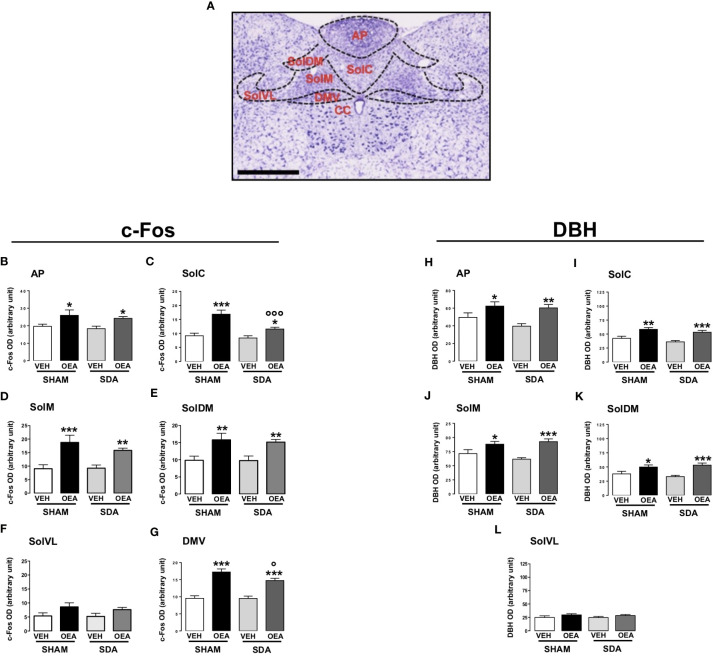
Patterns of c-Fos and DBH expression in the brainstem of both SHAM and SDA rats. Representative photomicrograph (scale bar = 500 μm) showing Nissl staining of coronal rat hindbrain section. The superimposed diagrams show the different subnuclei of the nucleus of the solitary tract (NST), such as commissural part (SolC), medial part (SolM), dorsomedial part (SolDM) and ventrolateral part (SolVL), the dorsal motor nucleus of the vagus (DMV) and the central canal (CC) **(A)**. Semiquantitative densitometric analysis of c-Fos (n=3-7 per group) **(B–G)** and of dopamine beta-hydroxylase (DBH) (n=4-6 per group) expressions **(H–L)** within the AP, SolC, SolM, SolDM, SolVL and DMV of both SHAM and SDA rats, treated with either vehicle (VEH; saline solution, PEG, Tween 80, 90/5/5 v/v/v; 2 ml kg^-1^) or OEA (10 mg kg^-1^, i.p.). Data are expressed as mean ± SEM. *p<0,05; **p<0,01; ***p<0,001 vs VEH in the same surgery group (Tukey’s test); °p<0,05; °°°p<0,001 vs SHAM in the same treatment group (Tukey’s test).

The patterns of c-Fos and DBH expression were evaluated at the level of the following brainstem nuclei: the AP, the dorsal motor nucleus of the vagus (DMV) and the NST, within its different subnuclei (schematically represented in **Figure 1A**), namely the commissural (SolC), the medial (SolM), the dorsomedial (SolDM), and ventrolateral (SolVL) parts.

**Figure 2:**
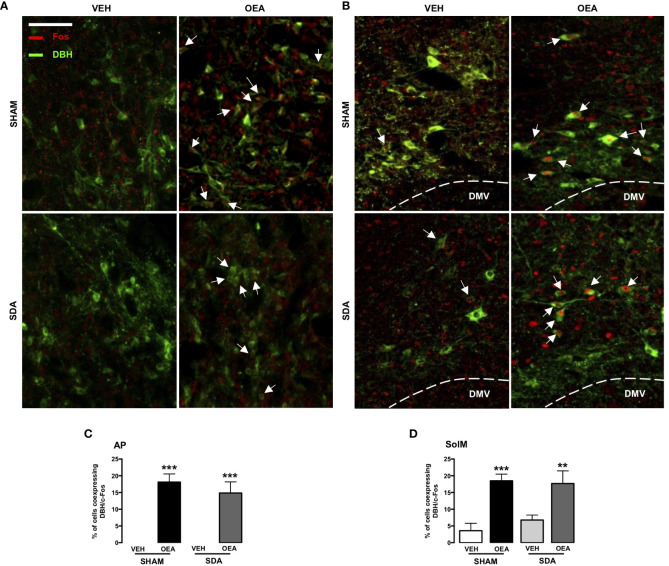
c-Fos and DBH double immunostaining within the AP and SolM of both SHAM and SDA rats. Representative fluorescent photomicrographs (x20 magnification, scale bar = 100 mm) showing c-Fos/dopamine beta-hydroxylase (DBH) double-immunostaining (red/green), of the area postrema (AP; **A**) and the medial part of nucleus of the solitary tract (SolM; **B**). White arrows indicate neurons, within AP and SolM, that co-express c-Fos and DBH signals (% of Fos-positive neurons: percentage of cells co-expressing c-Fos and DBH), measured in AP sections **(C)** and SolM sections **(D)** of SHAM and SDA rats after VEH or OEA treatment. Data are expressed as means ± SEM. ***p < 0.001 and **p < 0.01 vs. VEH in the same surgery group (Tukey’s test).

Based on our previous study reporting that the noradrenergic pathway from NST to PVN plays a necessary role in the pro-satiety effect of OEA (13), a second series of sections containing AP and SolM was double-stained for c-Fos and DBH (**Figure 2**) to qualitatively assess their colocalization within these brainstem structures.

**Figure 3:**
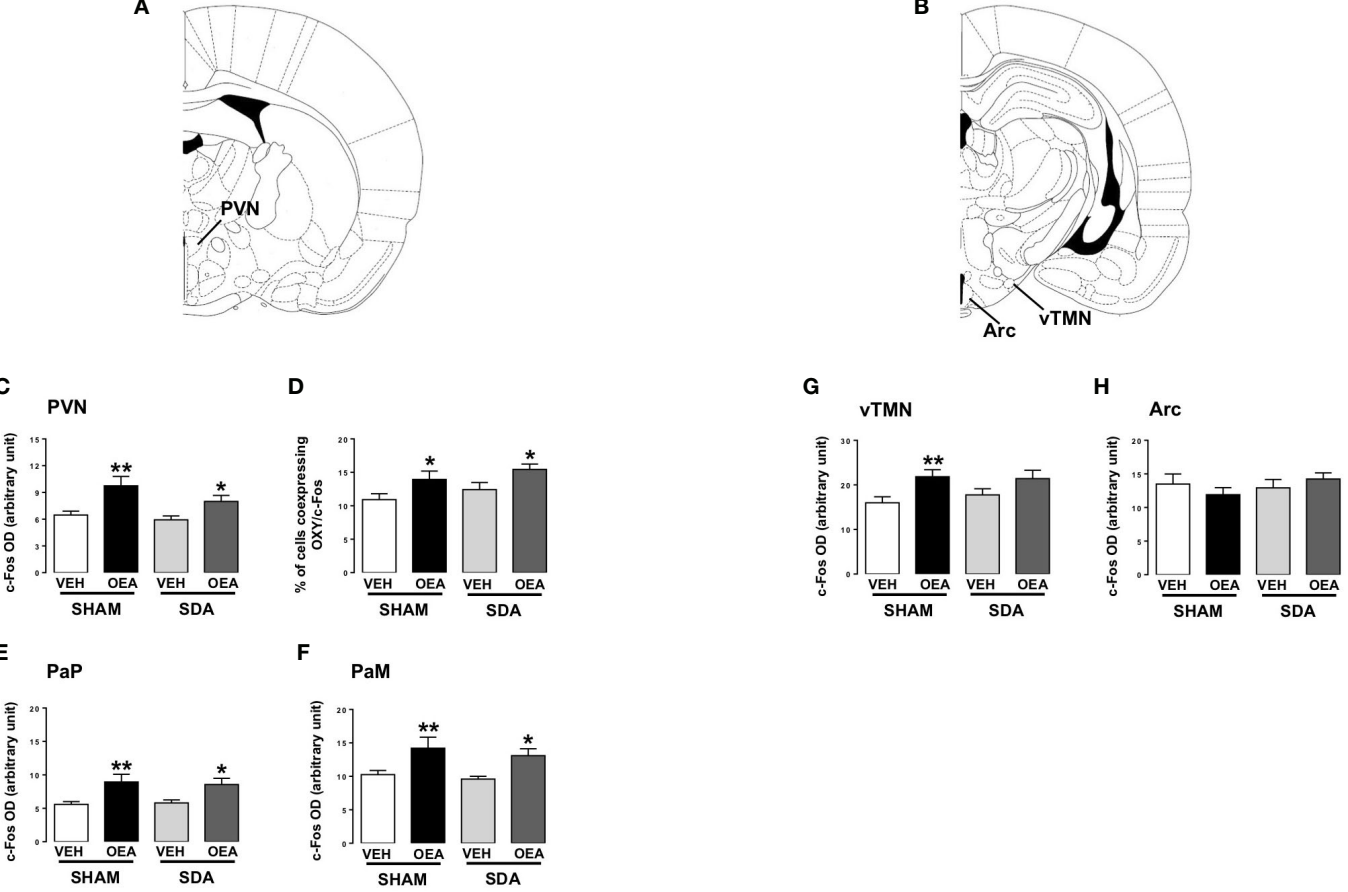
c-Fos expression in hypothalamic nuclei of both SHAM and SDA rats. Rat brain diagram taken from Paxinos brain atlas showing different hypothalamic nuclei **(A, B)**. Semiquantitative densitometric analysis of c-Fos (n=3-7 per group) expression within the paraventricular nucleus (PVN; **C**) parvocellular (PaP; **E**) and magnocellular (PaM; **F**) oxytocinergic neurons of the PVN, within the ventral part of the tuberomammillary nucleus (vTMN; **G**) and arcuate (Arc; **H**) nuclei and percentage of cells co-expressing c-Fos and oxytocin (OXY; **D**) of both SHAM and SDA rats, treated with either vehicle VEH or OEA (10 mg kg^-1^, i.p.). Data are expressed as mean ± SEM. *p<0,05; **p<0,01 vs VEH in the same surgery group (Tukey’s test).

Furthermore, c-Fos expression was specifically analyzed in the following hypothalamic nuclei well known for their role in energy homeostasis and eating-related motivation: PVN, arcuate nucleus (Arc) and the ventral TMN (vTMN) (**Figures 3A, B** show the rat brain diagram adapted from Paxinos brain atlas (43) reporting these nuclei). Finally, by double immunofluorescence, the co-expression of c-Fos and OXY in the PVN was also evaluated.

All the immunohistochemical analyses mentioned were performed by investigators blind to the different experimental groups and according to the protocols described in our previous work (13, 14, 17). We provide the detailed protocols again in the section **Supplementary Materials and Methods** of **Supplementary Material**.

##### 2.2.1.3 Semiquantitative analyses

All the brain sections were observed under a Nikon Eclipse 80i Advanced Research Microscope (RRID : SCR_015572) equipped with a color charge-coupled device camera and controlled by the software NIS-Elements Basic Research (RRID : SCR_002776). The DAB immunostaining was measured semi-quantitatively as optical density (OD) by using the program ImageJ (RRID : SCR_003070) and considering, for background normalization, the averaged OD either of non-immunoreactive regions or of white matter structures within the same brain slice according to our previous studies (13, 17).

The analyses of double immunofluorescence c-Fos/OXY, were conducted manually by counting separately each c-Fos- or OXY-positive cell of the PVN. Co-expression was assessed as the percentage of OXY-positive cells within c-Fos-positive neurons by following our previous analyses (13, 17).

In all the cases, measurements were obtained in at least four consecutive tissue sections containing the desired structure in at least 3 rats per experimental group. We used a brain atlas (43) to define localization of brain structures.

### 2.3 Experiment 2

#### 2.3.1 Brain pattern of OEA distribution and OEA plasma levels

##### 2.3.1.1 Animals and housing

Male Wistar-Han rats (Janvier Labs, Le Genest-Saint-Isle, France, n=80), weighing 275–325 g upon arrival, were individually housed in wire mesh cages under a 12:12 dark-light cycle in a climate-controlled room (22 ± 2°C and 60% relative humidity) and received *ad libitum* standard chow pellets (Provimi KIiba, Gossau, Switzerland) and water. All experiments were performed upon the approval of the Veterinary Office of the Canton of Zurich and according to the European Community directives 2010/63/EU. The rats were accustomed to handling and injections for 7 days before the beginning of the experiments. On the day of the experiment, food was temporarily removed from the cage 1 h before the beginning of the dark phase. At dark onset animals were injected with either OEA (10 mg kg^-1^ i.p.) or VEH and pre-weighed standard chow pellets were returned to the cages. At different time-points (2.5, 5, 15, 30, 60 min), rats were deeply anesthetized with isoflurane and blood was withdrawn from the heart and collected in glass tubes pre-coated with K_3_-EDTA and centrifuged at 4°C for plasma separation. Rats were then immediately sacrificed by decapitation and different brain areas of interest were freshly micro-dissected and snap frozen in liquid nitrogen until processing by UPLC-MS/MS analyses. The effect of OEA on food intake of these rats was also evaluated at the different time-points of sacrifice by manually measuring the difference between the amount of food given immediately after the treatment and the food remaining after each time point with correction for spillage. We focused our attention on the following brain areas: AP, NST, Arc/median eminence (ME) and hippocampus (HIPPO) known to be activated by OEA and to be involved in the control of both homeostatic and non-homeostatic eating (4, 13, 14, 16, 24, 44, 45). We decided to refer to Arc/ME by virtue of the strict anatomical proximity of these two brain areas that were collected during the fresh microdissection of the brain.

##### 2.3.1.2 UPLC-MS/MS analyses

By using UPLC-MS/MS analyses, we measured plasma and tissue content of NAEs (including OEA, PEA, AEA, SEA and LEA) and 2-AG in specific brain areas collected at 2.5, 5, 15, 30 and 60 min after systemic OEA (10 mg kg^-1^) administration. Briefly, frozen brain areas were homogenized in the presence of internal deuterated standards (5 pmol of d4-OEA, d4-AEA, d4-PEA, d4-SEA and 15 pmol of d5-2-AG), and then subjected to the lipid extraction procedure as previously described (46). The dry residue obtained was reconstituted with 30 μL of methanol. Plasma samples were extracted with the same procedure, by adding 100 μL of sample in each extraction vial. Briefly, the resulting lipid fractions were quantified according to the protocol used in previous studies (46, 47), by using an UPLC system coupled to a Xevo-TQ-S Mass Spectrometer (Waters Corporation, Milford, Massachusetts, USA). Analyte separation was achieved by using an Acquity UPLC BEH C18 column (1.7 μm, 2.1 × 50 mm; Waters Corporation) connected to an in-line filter unit and maintained at 40°C. Mobile phases A and B were composed of methanol-water-acetic acid (75:24.9:0.1, v/v/v) and methanol-acetic acid (99.9:0.1, v/v), respectively. The injection volume was 1 μl and the gradient 0.2 mL/min. An electrospray ionization source in positive mode was used. For data acquisition and processing, the software MassLynx (RRID : SCR_014271) was used. For each compound of interest, the ratio between the analyte and the respective internal standard was determined (**Figure S1** of **Supplementary Material**). Calibration curves were established by preparing standard solutions, which were diluted to obtain at least 10 calibration points, in the appropriate range for each compound. The amounts of each compound were determined by linear interpolation from the calibration curve. Data were analyzed by normalizing the pmol amount of each compound to the weight of the brain tissue samples (pmol/g) or to the volume of plasma samples (pmol/ml). All these analyses were conducted by investigators who were blind to the different experimental groups.

### 2.4 Statistical analyses

#### 2.4.1 Experiment 1

The immunochemical results were statistically analyzed by two-way ANOVA, with “surgery” and “treatment” as the two factors followed by Tukey’s *post-hoc* test to perform multiple comparisons.

Moreover, because of the difference in the number of slices examined and the high degree of freedom, the error degrees of freedom were kept constant at a value based on the actual number of animals per group (17).

#### 2.4.2 Experiment 2

Data obtained by UPLC-MS/MS analyses were statistically analyzed by two-way ANOVA with “time” and “treatment” as the two factors. Tukey’s test was used as a *post-hoc* to perform multiple comparisons.

Behavioral data were statistically analyzed by Student’s t-test with Bonferroni’s correction for multiple comparison between VEH-and OEA-treated animals for each time point considered (2.5, 5, 15, 30, 60 min).

All statistical analyses were carried out by using the software IBM SPSS Statistics (RRID : SCR_016479). In all instances, the threshold for statistical significance was set at p<0,05.

## 3 Results

### 3.1 Experiment 1

#### 3.1.1 Role of ascending subdiaphragmatic vagal fibers in OEA-induced activation of selected brain nuclei

In the present study we expanded our previous observation to examine whether SDA surgery abolishes the capability of OEA to activate selected brain nuclei and to affect noradrenergic and oxytocinergic neurotransmission.

The results from this experiment demonstrated that subdiaphragmatic vagal afferent fibers are not necessary for the activating effect of OEA on brain areas and its modulating effects on the noradrenergic and oxytocinergic systems.

In fact, semiquantitative densitometric analyses revealed that SDA surgery did not prevent the OEA-induced increases of c-Fos and DBH expression in all the brainstem nuclei analyzed (**Figures 1B–L**). Particularly, two-way ANOVA analyses followed by *post hoc* tests for multiple comparisons (Tukey’s test) revealed that OEA significantly increased the expression of both c-Fos and DBH at the level of the NST and its subnuclei, AP and DMV of both SHAM and SDA rats (Tukey’s results: p<0,05, p<0,01, p<0,001 vs VEH; **Figures 1B–E, G, H–K**). One exception is the SolVL where neither c-Fos nor DBH expression were affected by OEA treatment (**Figures 1F, L**) in both surgical groups. Interestingly, the increase of c-Fos expression induced by OEA in SDA rats was slightly attenuated at the level of the SolC (+38%) and the DMV (+55%), as compared to the respective increase observed in SHAM rats treated with OEA (+84% for SolC and +80% for DMV), (**Figures 1C, G**, p<0,001 and p<0,05 respectively). Two-way ANOVA results are reported in **Supplementary Material** in **Table S1**.

By analyzing the co-expression of both c-Fos and DBH at the level of SolM and AP, we found that SDA surgery did not prevent the capability of OEA to induce c-Fos expression in a group of noradrenergic neurons of both areas (**Figures 2A, B**) in keeping with our previous findings (17).

Moreover, semiquantitative densitometric analyses revealed that SDA surgery did not prevent OEA-induced increase of c-Fos expression in the PVN (**Figure 3C**, p<0,01 and p<0,05 vs respective VEH). In keeping with our previous results (13), OEA significantly increased the percentage of OXY-positive cells within c-Fos-positive ones in both SHAM and SDA rats (p<0,05 vs VEH; **Figure 3D**) without any difference between magnocellular and parvocellular components (**Figures 3E, F**, p<0,05 and p<0,01 vs respective VEH).

Finally, OEA increased c-Fos expression in the vTMN of SHAM animals (p<0,01 vs VEH; **Figure 3G**) while no significant effect was observed in SDA rats, thus suggesting a possible necessary role of vagal afferents in mediating OEA’s effects on histaminergic neurons in this area. Conversely, no significant difference between SHAM and SDA rats was observed at the level of the Arc (**Figure 3**), thus confirming previous findings (24).

Two-way ANOVA results are reported in **Supplementary Material** in **Table S1**.

### 3.2 Experiment 2

#### 3.2.1 Brain pattern of OEA distribution and OEA plasma levels

The results from this experiment demonstrated that an acute i.p. treatment with OEA, which rapidly inhibited food intake in rats, was associated with an equally fast increase of OEA levels in both plasma and in specific brain areas.

**Figure 4:**
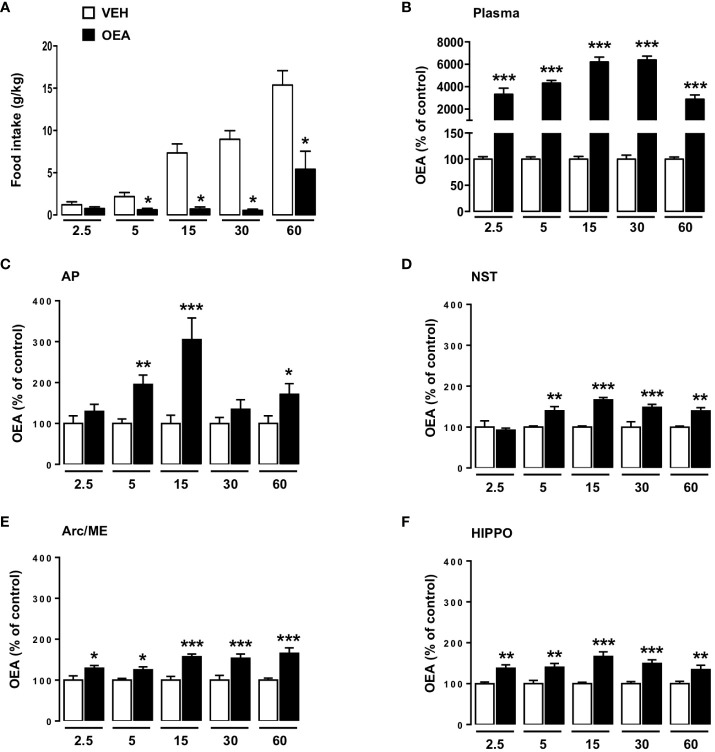
Effects of peripheral OEA administration on food intake, plasma and brain distribution of OEA at different time points. Food intake (normalized to body weight, g/kg; **A**) of rats sacrificed at different time points (2.5, 5, 15, 30, 60 min), treated with either vehicle VEH or OEA (10 mg kg^-1^, i.p.) (n=8 per group). Data are expressed as mean ± SEM. *p<0,05 vs VEH at the same time point (Student’s t-test with Bonferroni’s correction for multiple comparisons). OEA levels expressed as % with respect to the average levels observed in VEH treated animals (controls) sacrificed at the same time-point considered for OEA treatment. Data refers to plasma **(B)**, area postrema (AP; **C**), nucleus of the solitary tract (NST; **D**), arcuate nucleus/median eminence (Arc/ME; **E**) and hippocampus (HIPPO; **F**) of rats sacrificed at different time points (2.5, 5, 15, 30, 60 min) after acute administration of OEA (10 mg kg^-1^, i.p.) or vehicle (VEH). Data are expressed as mean ± SEM. * p<0,05, ** p<0,01 *** p<0,001 vs VEH in the same time point (Tukey’s test; n=6-8).

In particular, the results showed that OEA treatment significantly decreased food intake at 5, 15, 30 and 60 (p<0,05) min after its i.p. injection. No significant reduction in food intake was observed at the 2.5 min time point (**Figure 4A**). Moreover, two-way ANOVA performed on data obtained by UPLC-MS/MS analyses revealed that plasma OEA levels rose as early as 2.5 min after i.p. injection (p<0,001 vs respective controls, **Figure 4B**) and remained elevated until 1 h, with a peak increase registered at 15-30 min (about 60-fold higher than controls). Two-way ANOVA results are reported in **Supplementary Material** in the **Table S2**.

The results regarding plasma AEA, PEA, LEA, SEA and 2-AG concentration are reported in the section **Supplementary Results** and **Tables S3, S4** of **Supplementary Material**. These results demonstrated that all the NAEs were increased in the plasma of OEA-treated animals, whereas 2-AG levels remained unaffected.

Concerning the brain distribution of OEA, our results suggest that OEA reached the brain and permeated all the areas analyzed in a few minutes after its administration. Moreover, its level remained significantly elevated until 1 h (**Figures 4C–F**). In particular, two-way ANOVA analyses revealed that already at 2.5 min after injection the OEA content was significantly higher than after VEH injection at the level of the Arc/ME (p<0,05), and HIPPO (p<0,01) (**Figures 4E, F** respectively), whereas such an effect appeared slightly later (5 min time point) at the level of the AP and NST (**Figures 4C, D**; p<0,01).

The maximum increase of OEA brain distribution was registered 15 min after its administration in almost all the brain areas analyzed, with the highest increase observed within the AP (**Figures 4C, D, F**; p<0,001), except for the Arc/ME where the highest increase was observed at the 60 min time point (**Figure 4E**; p<0,001). Of note the levels of the other NAEs measured in the brain were not altered by OEA administration.

Two-way ANOVA results are reported in **Supplementary Material** in the **Table S2**.

The results obtained on brain NAEs and 2-AG levels are reported in the section **Supplementary Results** and **Tables S3, S4** of **Supplementary Material**.

## 4 Discussion

Our results demonstrate, for the first time, that OEA is distributed in the brain as an intact molecule (**Figure S1** of **Supplementary Material**) within a few minutes after its acute i.p. injection. Indeed, the time-course measurements performed in the present study suggest a rapid OEA permeation of specific brain areas, partaking in the circuits controlling eating behavior and metabolism and partially known for being involved in the eating-inhibitory effect of systemically administered OEA. Such areas include the AP and the NST in the brainstem, the hypothalamic Arc, the ME and the HIPPO.

Interestingly, OEA concentrations reached the highest increase at the level of the AP, where they were up to 3-fold higher in OEA- than in VEH-treated rats (305.3 ± 52.87 vs 100 ± 20.30 respectively) as compared to the other brain areas analyzed (Arc/ME, NST, and HIPPO), where OEA levels were only about 1.5-fold higher than in controls (Arc/Me: 157.1 ± 6.6 vs 100 ± 9.2; NST: 166.6 ± 5.6 vs 100 ± 2.7; HIPPO: 166.5 ± 11.3 vs 100 ± 3.6). These results suggest that the AP might be an important part of the central circuits involved in mediating OEA’s pharmacological effects, thus corroborating our previous findings of the necessary role of this area in mediating OEA effects on eating (17). Moreover, the observation that OEA reached high levels also in the NST starting at the same time as in the AP (5 min) suggests that, once permeated into the AP, OEA rapidly diffuses into the anatomically adjacent NST. Moreover, in the present study we also considered the Arc/ME and HIPPO because of their high density of PPAR-α (48, 49), the main receptor responsible for the eating-inhibitory effect of OEA (35). Here, we demonstrate for the first time that intact OEA physically reaches these brain areas already at 2.5 min after its i.p. administration. In our previous studies in freely eating rats OEA did not induce *c-fos* mRNA at the level of the Arc, nor did it change the expression of proopiomelanocortin, which is highly expressed in the Arc and generates peptide signals that control PVN and supraoptic nucleus activities (24). In contrast, Umehara et al. (50) observed that peripheral OEA increased the expression of c-Fos in the Arc of fasted wild type mice, without producing any effect in the Arc of histidine decarboxylase knock-out mice, suggesting a necessary role of histaminergic neurons in mediating c-Fos induction in the Arc by OEA (50). Presumably, the different species (mouse *vs* rat) used, and, even more importantly the different homeostatic states of the animals (fasted *vs* fed) are responsible for the divergent observations.

The increased OEA concentration observed at the level of the HIPPO at the first time point considered (2.5 min) is in line with several studies suggesting that the HIPPO represents the primary brain region receiving ligands from both the blood and the cerebrospinal fluid (51), by virtue of its anatomical position, lying alongside the choroid plexus with its rich blood supply and immediately adjacent to the cerebral ventricles (43, 51). Importantly, PPAR-α are expressed in the HIPPO (52), thus suggesting that this region might represent another direct target site for circulating OEA. The HIPPO is primarily recognized as the brain area implicated in learning and memory processes (52), and a variety of studies demonstrate an effect of exogenous OEA on cognitive features. In fact, it has been demonstrated that OEA controls short-term spatial memory and hippocampal neurogenesis (53). Likewise, Yang et al. (33) demonstrated that systemic OEA improves spatial cognitive deficits in a rat model of acute cerebral ischemic injury through enhancing neurogenesis in the HIPPO (33). Finally, we recently demonstrated that OEA exerts antidepressant-like effects in mice and that this is associated with an increased expression of hippocampal phospho- cAMP response element-binding protein, a feature common to other PPAR-α agonists with antidepressant-like effects (54, 55). Interestingly, it has been recently demonstrated that eating behavior might be influenced by hippocampal-dependent memory functions, including meal-related memories and conditional learned associations between food and post-ingestive effects [for review see (56)]. We therefore hypothesize that by binding to PPAR-α in the HIPPO, OEA might modulate memory processes both related to food and cognition.

Although previous studies suggested that OEA is ineffective in controlling eating after its intracerebroventricular injection (4), the present results indicate that the eating-inhibitory effect of OEA is paralleled by an OEA distribution in the brain that is consistent with the assumption of its direct effects in one or more brain areas. These controversial findings may be explained by the high expression of fatty acid amide hydrolase in the brain ventricular epithelium (57), which might limit the bioavailability of OEA in brain tissue after intracerebroventricular administration and, hence, explain its ineffectiveness *via* this route of administration. Future studies should address this possibility.

Although brain OEA levels were already increased at 2.5 min after its i.p. administration, the maximum increase was observed at the 15-min time-point in most of the areas considered. This observation fits the behavioral data showing the greatest inhibitory effect of OEA on eating at 15-30 min after administration. The food intake reducing effect of i.p. injected OEA might therefore reflect its concentration in the brain. This interpretation is consistent with our previous observations demonstrating the necessary role of key brain sites in mediating OEA’s behavioral actions (13, 14, 16–18, 58) and indicates that such areas are recruited by OEA directly, rather than being activated as second- or third-order circuits downstream of peripheral nerves.

A previous study reported OEA brain levels in rats after its acute oral administration (59) showing no increase as compared to vehicle-treated animals. However, this discrepancy is not surprising for several reasons. First, compared to the present investigation, in that study orally administered OEA presumably underwent substantial first-pass hepatic extraction, which most likely limited the bioavailability of the molecule significantly. Second, the analyses were performed on the total brain, without any brain area separation, which most likely missed possible increases of OEA levels in specific brain areas. Third, rats were sacrificed at later time points, which is presumably unsuited to catch possible differences in OEA brain content following an acute systemic administration, due to the rapid permeation of the molecule into the brain and its subsequent hydrolysis. These latter two issues, namely the analyses in the whole brain and late time-points might explain also the negative results on brain distribution of OEA reported in a recent study (75) based on the intravenous administration of deuterated-OEA (^13^C-OEA) to rats at the dose of 1 mg/kg.

In parallel to investigating OEA brain distribution, in the present study we also measured OEA plasma levels at the same time points. Our results demonstrate that OEA plasma levels significantly increase as early as 2.5 min (30-fold higher than control, 3,333 ± 531 vs 100 ± 5) after its i.p. injection, with a maximum increase registered at 15-30 min (60-fold higher than controls respectively 6,218 ± 424 vs 100 ± 5 and 6,394 ± 344 vs 100 ± 8). The combination of the OEA distribution data in brain and plasma indicates that, once administered, OEA acts as a humoral mediator rapidly reaching brain structures including the Arc/ME, AP and HIPPO, which allow not only its access to the brain, but also likely represent brain target sites by virtue of the presence of PPAR-α, the major receptor for OEA. Beyond the HIPPO characteristics already described above, it is important to mention that both AP and Arc/ME are sensory circumventricular midline structures located around the fourth and third ventricles, respectively, lacking a blood brain barrier, with neurons of both areas being more exposed to blood-borne agents (60). This is in line with previous studies suggesting that the AP and Arc/ME represent easy access sites for circulating endogenous and exogenous eating-relevant hormones including amylin, glucagon-like peptide-1 and ghrelin (61, 62). Interestingly, very recent evidence from the research team led by Dr D’Agostino (63), demonstrates that systemic administration of OEA (10 mg/kg) to food-deprived female mice produces a rapid and stable inhibition of Agouti-related protein positive neurons of the Arc, which are strongly activated during a negative energy balance to stimulate eating. In their (63) OEA’s inhibitory effect on these neurons was maximal at 5 min after i.p. administration, thus mimicking the time course of OEA brain distribution observed in our experiment.

The results obtained in Experiment 1, i.e., that the OEA-induced activation of brainstem and hypothalamic nuclei did not depend on intact subdiaphragmatic vagal afferents, is consistent with our previous behavioral results (15), and further supports the view that OEA may act as a blood-borne messenger. Interestingly, also the effects of OEA on the brainstem noradrenergic and the PVN oxytocinergic systems were not affected by SDA, as OEA still maintained its capability to increase the expression of DBH in the brainstem nuclei and the activation of OXY neurons in the PVN in SDA rats. The only nucleus analyzed that seems to be under the control of afferent fibers is the vTMN because SDA prevented the activation of this nucleus by OEA, also indicating that OEA does affect vagal afferent signaling.

Interestingly, a communication between the TMN and the NST has been described; in particular, histaminergic neurons of the TMN extend their fibers to the NTS (64, 65). However, there is no direct evidence suggesting that such input affects eating. The slight attenuation of the OEA-induced c-Fos expression in the SolC and DMV of SDA rats might be, at least in part, the consequence of the lacking activation of vTMN in the same animals, although future studies are needed to address this assumption. However, the significant increase observed anyway at both sites might be associated with the activity of still intact efferent vagal fibers projecting from the brainstem to the gut, already known to be involved in modulating other satiety/appetite-signals, such as CCK (66). This result might partially explain the loss of OEA’s eating-inhibitory effect observed in rats (35) after total subdiaphragmatic vagotomy and the failure of the SDA procedure to abolish this behavioral effect of OEA.

Perhaps the inhibition of eating exerted by OEA results from a combination of both, humoral and vagal pathways, such that the short-term effect on eating is due to the direct activation of brain nuclei shown here, whereas the sustained effects on satiety (as suggested by the lack of compensatory overfeeding) may be due to the subsequent activation of vagal efferents, which might relay an OEA signal from the brain to the gut. Future studies might address this hypothesis.

Finally, as both the biosynthetic and catabolic pathways of OEA are shared by other NAEs (2), we also investigated whether the OEA treatment might affect the levels of other NAEs, and 2-AG in both plasma and brain. Interestingly, while AEA was the only endogenous compound affected by OEA in the brain (decreased level in the HIPPO), in the plasma all the NAEs analyzed were increased except for 2-AG that was unaffected. Because AEA is well known to stimulate appetite by activating CB1 receptors (67), its decrease in the HIPPO might contribute to the eating-inhibitory effect of OEA. However, future studies are necessary to examine this possibility. Concerning the increased plasma level of NAEs, we might refer to the theory of the ‘entourage’ effect (68, 69). According to this concept, peripherally administered OEA might cause an accumulation of endogenous NAEs by preventing their degradation, due to the saturation of the hydrolyzing pathways, which are shared among the different NAEs. Although OEA has been found to be the most active NAE in reducing energy intake, evidence suggests that also other NAEs, such as PEA, might participate in this process (67, 70, 71). Therefore, we cannot exclude that circulating NAEs might contribute to OEA’s pharmacological effects both related or not to eating and metabolism.

However, these aspects were beyond the aim of the present work, and future studies are needed to address these points.

## 5 Conclusions

In conclusion, the results obtained in the present study describe for the first time that exogenous OEA is able to distribute in the brain very rapidly after its administration. The timeline of this phenomenon parallels OEA’s hypophagic action. Moreover, OEA does not require intact vagal afferents from below the diaphragm to exerts the central neurochemical effects associated to its hypophagic action. Taken together these observations indicate that OEA can modulate eating behavior by directly targeting neurons of specific brain nuclei.

## Data availability statement

The raw data supporting the conclusions of this article will be made available by the authors, without undue reservation.

## Ethics statement

The animal study was reviewed and approved by Veterinary Office of the Canton of Zurich and according to the European Community directives 2010/63/EU.

## Author contributions

Conceptualization, AR, SG, WL, TL, and GGM. Data curation, AR, MF, AP, and GGM. Formal analysis, MF, BE, CG, AP, and JK. Funding acquisition, AR and SG. Investigation, MF, BE, CG, JK, and EA. Methodology, MF, BE, CG, JK, EA, MA, AP, and GGM. Project administration, AR and SG. Supervision, AR, SG, WL, TL, and GGM. Validation, AR and SG. Writing - original draft, MF and AR. Writing, review, and editing, SG, WL, AR, TL, and GGM. All authors contributed to the article and approved the submitted version.
