# Supplementary material for: “To brain or not to brain”: evaluating the possible direct effects of the satiety factor oleoylethanolamide in the central nervous system
Source: Front Endocrinol (Lausanne). 2023 May 10;14:1158287. doi: 10.3389/fendo.2023.1158287 (PMC10206109; doi:10.3389/fendo.2023.1158287)
Supplement: Supplementary file 1 [file DataSheet_1.docx]

Supplementary Material

“To brain or not to brain”: evaluating the possible direct effects of the satiety factor oleoylethanolamide in the central nervous system

Adele Romano^1†*^, Marzia Friuli^1†^, Barbara Eramo^1^, Cristina Anna Gallelli^1^, Justyna Barbara Koczwara^1^, Elnaz Karimian Azari^2,††^, Adrien Paquot^3^, Myrtha Arnold^2^, Wolfgang Langhans^2^, Giulio Giorgio Muccioli^3^, Thomas Alexander Lutz^4^ and Silvana Gaetani^1^

^1^Department of Physiology and Pharmacology “V. Erspamer”, Sapienza University of Rome, Rome, Italy

^2^Physiology and Behavior Laboratory, ETH Zurich, Zurich, Switzerland

^3^Bioanalysis and Pharmacology of Bioactive Lipids Research Group, Louvain Drug Research Institute, Université catholique de Louvain, Louvain-la-Neuve, Belgium

^4^Institute of Veterinary Physiology, Vetsuisse Faculty, University of Zurich, Zurich, Switzerland

**^†^** These authors contributed equally to this work and share first authorship

*** Correspondence:**Adele Romano
[adele.romano@uniroma1.it](mailto:adele.romano@uniroma1.it)

**^††^** Current affiliation: Lonza Greenwood LLC, 535 Emerald Rd N, Greenwood, SC 29646, Stati Uniti

**Supplementary Materials and Methods**

## Experiment 1: Immunohistochemical studies

For immunohistochemical experiments brains from both SHAM and SDA rats administered with either vehicle (VEH) or OEA (10 mg kg^-1^) were cut on a cryostat (model HM550; Thermo Fisher Scientific, Kalamazoo, MI, USA) into five series of 20-μm coronal sections containing hypothalamic and brainstem structures of interest and mounted on positively charged microscope slides (SuperFrost Plus, Menzel, Germany) and stored at −20°C until processed.

***c-Fos and DBH chromogenic immunostaining***

We followed our previous studies for c-Fos and DBH chromogenic immunostaining [1,2]. Briefly, sections were rehydrated in PBS (pH 7.4) and then incubated for 1.5 h in a solution containing 2% normal donkey serum (Jackson ImmunoResearch Labs Cat# 017-000-121, RRID:AB_2337258) in 0.1% Triton X-100 (Sigma-Aldrich; PBST), followed by incubation with the primary antibody (rabbit polyclonal anti-c-Fos primary antibody Santa Cruz Biotechnology Cat# sc-52, RRID:AB_2106783, 1:5000 or mouse monoclonal anti-DBH primary antibody, Millipore, Cat# MAB308, RRID: AB_2245740, 1:1000) at 4°C, overnight. Sections were then incubated with the secondary antibody (donkey anti-rabbit biotinylated secondary antibody, Jackson ImmunoResearch Labs Cat# 711-067-003, RRID:AB_2340595, 1:500 or donkey anti-mouse biotinylated secondary antibody, Jackson ImmunoResearch Labs Cat# 715-065-150, RRID:AB_2307438, 1:400) in 0.1% PBST for 2h at room temperature. After incubation for 1h with the ABC Kit (Vector Laboratories Cat# PK-6100, RRID:AB_2336819), sections were stained by incubation in DAB (Vector Laboratories Cat# SK-4105, RRID:AB_2336520) chromogen solution. The slides were then rinsed with PBS, dehydrated in graded alcohol, immersed in xylene and cover-slipped with Eukitt (Sigma-Aldrich). In every step of the protocol, all sections from all the experimental groups were processed together at the same time, to ensure the same experimental conditions to each sample and reliable densitometric analyses.

***c-Fos and DBH double fluorescence immunostaining***

For double fluorescent immunostaining for c-Fos and DBH we followed the protocol adopted in our previous study [3].

Briefly, sections were rinsed with PBS and incubated for 1 h in a solution containing 0.3% Triton X-100 (Sigma-Aldrich) and 2% of normal goat serum (Jackson ImmunoResearch Labs Cat# 005-000-121, RRID:AB_2336990) in PBS. Sections were then incubated with the primary antibodies (rabbit polyclonal anti-c-Fos primary antibody Santa Cruz Biotechnology Cat# sc-52, RRID:AB_2106783, 1:500; mouse monoclonal anti-DBH primary antibody, Millipore, Cat# MAB308, RRID: AB_2245740, 1:1000) for 2 overnights at 4 °C. Sections were then incubated with the secondary antibodies (goat anti-rabbit Alexa Fluor 594 secondary antibody Thermo Fisher Scientific Cat# A-11012, RRID:AB_2534079, 1:300; goat anti-mouse Alexa Fluor 488 secondary antibody Thermo Fisher Scientific Cat# A-11001, RRID:AB_2534069, 1:250) for 90 min at room temperature in the presence of Hoechst 33258 (Sigma-Aldrich, 1:5000), used to detect cell nuclei. After final washes, slides were cover-slipped with Fluoromount (Sigma-Aldrich).

***c-Fos and OXY double fluorescence immunostaining***

By following our previous study [2], after rehydration in PBS, brain sections were incubated in 2% normal goat serum (Jackson ImmunoResearch Labs Cat# 005-000-121, RRID:AB_2336990) in 0.3% Triton X-100 (PBST) for 1 h. Sections were then incubated for one overnight at room temperature with a rabbit polyclonal anti-c-Fos primary antibody (Santa Cruz Biotechnology Cat# sc-52, RRID:AB_2106783, 1:5000) and with a mouse monoclonal anti-OXY primary antibody (Millipore Cat# MAB5296, RRID:AB_2157626, 1:1000). Sections were then rinsed in PBST and incubated with a goat anti-mouse Alexa Fluor 488 (Thermo Fisher Scientific Cat# A-11001, RRID:AB_2534069, 1:400) and a goat anti-rabbit Alexa Fluor 594 (Thermo Fisher Scientific Cat# A-11012, RRID:AB_2534079, 1:300) secondary antibodies for 2 h. After additional washes, the sections were cover-slipped with Fluoromount (Sigma-Aldrich).

***Semiquantitative analyses***

All the brain sections were observed under a Nikon Eclipse 80i Advanced Research Microscope (RRID:SCR_015572) equipped with a color charge-coupled device camera and controlled by the software NIS-Elements Basic Research (RRID:SCR_002776). The DAB immunostaining was measured semi-quantitatively as optical density (OD) by using the program ImageJ (RRID:SCR_003070) and considering, for background normalization, the averaged OD either of non-immunoreactive regions or of white matter structures within the same brain slice. For the double immunofluorescence analyses, c-Fos- and OXY-positive cells were manually counted and the colocalization was assessed as the percentage of OXY-positive cells within c-Fos-positive neurons by following our previous analyses [2].

# Supplementary Results

**Experiments 2: Effects of OEA on plasma and brain distribution of other NAEs and 2-AG**

Two-way ANOVA analyses performed on data obtained from UPLC-MS/MS analysis revealed that OEA treatment significantly affected the levels of other NAEs in the plasma as compared to control animals, with different kinetics (Table S3 reports the two-way ANOVA output; Tukey post hoc analyses are reported in Table S4). In particular, the maximum increase in the amount of AEA (p<0,01), PEA (p<0,001) and LEA (p<0,001) was registered at 15 min after OEA treatment whereas SEA reached the maximum levels 30 min after OEA systemic administration (p<0,001; Table S4). Overall, no change was observed for 2-AG levels except for those measured at 15 min after OEA treatment; at such a time point was observed a significant reduction of 2-AG amount in OEA-treated rats compared to controls (p<0,01; Table S4).

Concerning the effect of OEA treatment on brain distribution of the other NAEs and 2-AG, the results obtained by two-way ANOVA revealed no change in all the brain areas analyzed at all the time points considered, except for AEA levels measured in the HIPPO after 30 min from OEA treatment; at such time point was observed a significant reduction of AEA amount in OEA-treated rats compared to controls (p<0,01). Table S3 reports the two-way ANOVA output, while Tukey post hoc analyses are reported in Table S4.

# Supplementary Bibliography

[1] G. Provensi, R. Coccurello, H. Umehara, L. Munari, G. Giacovazzo, N. Galeotti, D. Nosi, S. Gaetani, A. Romano, A. Moles, P. Blandina, M.B. Passani, Satiety factor oleoylethanolamide recruits the brain histaminergic system to inhibit food intake, Proc Natl Acad Sci U S A. 111 (2014). https://doi.org/10.1073/pnas.1322016111.

[2] A. Romano, C.S. Potes, B. Tempesta, T. Cassano, V. Cuomo, T. Lutz, S. Gaetani, Hindbrain noradrenergic input to the hypothalamic PVN mediates the activation of oxytocinergic neurons induced by the satiety factor oleoylethanolamide, Am J Physiol Endocrinol Metab. (2013). https://doi.org/10.1152/ajpendo.00411.2013.

[3] A. Romano, C.A. Gallelli, J.B. Koczwara, F.E. Braegger, A. Vitalone, M. Falchi, M.V. Micioni Di Bonaventura, C. Cifani, T. Cassano, T.A. Lutz, S. Gaetani, Role of the area postrema in the hypophagic effects of oleoylethanolamide, Pharmacol Res. (2017). <https://doi.org/10.1016/j.phrs.2017.05.017>.

# Supplementary Figures and Tables (If there are Supplementary Figures, please include the caption in the same file as the figure)


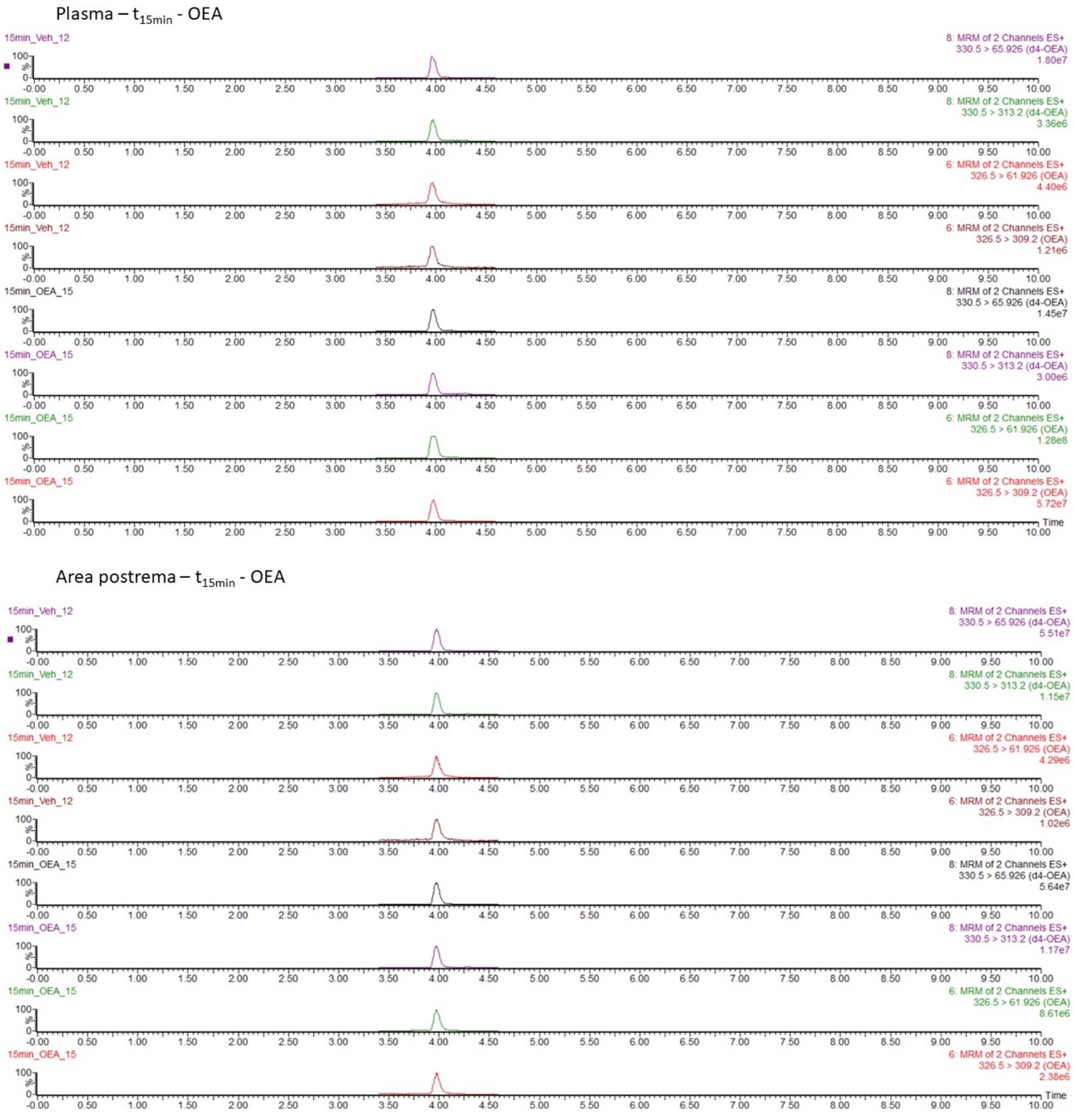


**Fig. S1 Chromatograms of the NAE-containing lipid fraction showing OEA peaks**

Representative chromatograms of the NAE-containing lipid fraction extracted from the plasma (upper panel) or area postrema (lower panel) of a VEH-treated rat (4 upper traces showing endogenous OEA) and of an OEA-treated rat (4 lower traces, showing endogenous + exogenous OEA) within the animals sacrificed 15 min after treatment. The traces show the extracted-ion chromatograms (EIC) for d4-OEA (quantitative transition 330.5-to-65.9 and qualitative transition 330.5-to-313.2) and the EIC for OEA (quantitative transition 326.5-to-61.9 and qualitative transition 326.5-to-309.2).

|  |  | **F treatment** | **F surgery** | **F interaction** | **df** |
| --- | --- | --- | --- | --- | --- |
| **c-Fos** | **AP** | **10,148 (p<0,01)** | 0,636 (p=0,436) | 0,012 (p=0,914) | 1/17 |
|  | **SolC** | **28,594 (p<0,001)** | **8,929 (p<0,01)** | **4,884 (p<0,05)** | 1/20 |
|  | **SolM** | **22,995 (p<0,001)** | 0,643 (p=0,433) | 0,852 (p=0,368) | 1/19 |
|  | **SolDM** | **16,687 (p<0,001)** | 0,069 (p=0,795) | 0,04 (p=0,843) | 1/19 |
|  | **SolVL** | **5,692 (p<0,05)** | 0,232 (p=0,636) | 0,121 (p=0,732) | 1/19 |
|  | **DMV** | **67,977 (p<0,001)** | 2,640 (p=0,121) | 2,496 (p=0,131) | 1/19 |
|  | **PVN** | **16,883 (p<0,001)** | 3,139 (p=0,090) | 0,865 (p=0,362) | 1/21 |
|  | **c-Fos^+^/OXY^+^** | **8,896 (p<0,01)** | 2,198 (p=0,152) | 0,000 (p=0,992) | 1/23 |
|  | **PaP** | **13,690 (p<0,01)** | 0,011 (p=0,918) | 0,138 (p=0,715) | 1/18 |
|  | **PaM** | **11,313 (p<0,01)** | 0,648 (p=0,431) | 0,040 (p=0,844) | 1/18 |
|  | **vTMN** | **9,438 (p<0,01)** | 0,194 (p=0,665) | 0,508 (p=0,486) | 1/15 |
|  | **Arc** | 0,013 (p=0,910) | 0.574 (p=0,458) | 1,535 (p=0,230) | 1/19 |
|  |  |  |  |  |  |
| **DBH** | **AP** | **13,178 (p<0,01)** | 1,721 (p=0,205) | 0,767 (p=0,383) | 1/19 |
|  | **SolC** | **27,68 (p<0,001)** | 3,541 (p=0,085) | 0,033 (p=0,857) | 1/22 |
|  | **SolM** | **19,52 (p<0,001)** | 0,242 (p=0,628) | 1,805 (p=0,1941) | 1/20 |
|  | **SolDM** | **16,49 (p<0,001)** | 0,035 (p=0,853) | 1,061 (p=0,315) | 1/20 |
|  | **SolVL** | 3,002 (p=0,098) | 0,097 (p=0,759) | 0,051 (p=0,824) | 1/20 |

**Table S1 Two-way ANOVA results of the patterns of c-Fos and DBH expression in different brain areas**

Results of the two-way ANOVA analyses of c-Fos and DBH expression observed in the AP, NST subnuclei, DMV and hypothalamus of both SHAM and SDA rats after intraperitoneal infusion of OEA or VEH. Area postrema (AP), commissural (SolC), medial (SolM), dorsomedial (SolDM) and ventrolateral (SolVL) parts of the NST, dorsal motor nucleus of the vagus (DMV), paraventricular nucleus (PVN), percentage of cells co-expressing Fos/OXY, parvocellular (PaP) and magnocellular (PaM) oxytocinergic neurons of the PVN, ventral part of the tuberomammilary (vTMN) and arcuate (Arc) nuclei of the hypothalamus.

|  |  | **F treatment** | **F surgery** | | **F interaction** | **df** |
| --- | --- | --- | --- | --- | --- | --- |
|  | **Plasma** | **645,049 (p<0,001)** | | **16,473 (p<0,001)** | **16,473 (p<0,001)** | 1/78 |
|  | **AP** | **29,330 (p<0,001)** | **3,800 (p<0,01)** | | **3,800 (p<0,01)** | 1/77 |
| **OEA** | **NST** | **49,027 (p<0,001)** | **4,822 (p<0,01)** | | **4,822 (p<0,01)** | 1/75 |
|  | **Arc/ME** | **67,384 (p<0,001)** | 2,032 (p=0,099) | | 2,032 (p=0,099) | 1/79 |
|  | **HIPPO** | **87,702 (p<0,001)** | 1,131 (p=0,274) | | 1,131 (p=0,274) | 1/79 |

**Table S2** **Two-way ANOVA results of plasma and brain distribution of OEA**

Results of the two-way ANOVA analyses of OEA distribution, in plasma and in different brain areas of rats sacrificed at different time points after intraperitoneal injection of either OEA (10 mg kg^-1^) or vehicle (VEH). Area postrema (AP), nucleus of the solitary tract (NST), arcuate nucleus/median eminence (Arc/ME), hippocampus (HIPPO).

|  |  | **F treatment** | **F time** | **F interaction** | **df** |
| --- | --- | --- | --- | --- | --- |
| **Plasma** | **OEA** | **635,248 (p<0,001)** | **12,713 (p<0,001)** | **12,866 (p<0,001)** | 1/78 |
|  | **AEA** | **15,847 (p<0,001)** | 2,334 (p=0,064) | **2,682 (p<0,05)** | 1/79 |
|  | **PEA** | **59,285 (p<0,001)** | **3,790 (p<0,01)** | 1,966 (p=0,110) | 1/77 |
|  | **LEA** | **290,397 (p<0,001)** | **11,309 (p<0,001)** | **11,804 (p<0,001)** | 1/78 |
|  | **SEA** | **419,094 (p<0,001)** | **41,454 (p<0,001)** | **30,815 (p<0,001)** | 1/78 |
|  | **2-AG** | 0,429 (p=0,515) | **2,667 (p<0,05)** | **2,935 (p<0,05)** | 1/79 |
|  |  |  |  |  |  |
|  | **OEA** | **23,702 (p<0,001)** | **5,724 (p<0,001)** | 1,653 (p=0,171) | 1/77 |
|  | **AEA** | 0,012 (p=0,912) | 0,368 (p=0,830) | 0,179 (p=0,948) | 1/78 |
| **AP** | **PEA** | 0,015 (p=0,904) | 0,255 (p=0,906) | 0,114 (p=0,977) | 1/74 |
|  | **LEA** | 0,003 (p=0,959) | 1,391 (p=0,248) | 0,108 (p=0,979) | 1/68 |
|  | **SEA** | 0,023 (p=0,879) | 0,380 (p=0,822) | 0,232 (p=0,920) | 1/73 |
|  | **2-AG** | 0,081 (p=0,777) | 1,601 (p=0,184) | 0,822 (p=0,561) | 1/78 |
|  | | | | | |
|  | **OEA** | **41,433 (p<0,001)** | **4,811 (p<0,01)** | **4,280 (p<0,01)** | 1/75 |
|  | **AEA** | 1,790 (p=0,186) | 1,528 (p=0,207) | 0,175 (p=0,950) | 1/64 |
| **NST** | **PEA** | 1,668 (p=0,201) | 2,240 (p=0,074) | 2,346 (p=0,063) | 1/76 |
|  | **LEA** | 0,298 (p=0,587) | **4,091 (p<0,01)** | 0,409 (p=0,801) | 1/74 |
|  | **SEA** | 0,006 (p=0,941) | 1,106 (p=0,361) | 1,418 (p=0,238) | 1/74 |
|  | **2-AG** | **8,306 (p<0,01)** | **2,774 (p<0,05)** | 0,429 (p=0,788) | 1/77 |
|  |  |  |  |  |  |
|  | **OEA** | **65,676 (p<0,001)** | **5,036 (p<0,01)** | 1,793 (p=0,140) | 1/79 |
|  | **AEA** | 0,650 (p=0,423) | 1,730 (p=0,156) | 0,416 (p=0,797) | 1/67 |
| **ME** | **PEA** | 1,325 (p=0,254) | 0,893 (p=0,473) | 1,394 (p=0,245) | 1/77 |
|  | **LEA** | 2,667 (p=0,107) | 0,364 (p=0,834) | 0,402 (p=0,807) | 1/74 |
|  | **SEA** | 0,000 (p=0,987) | **8,132 (p<0,001)** | 1,563 (p=0,195) | 1/75 |
|  | **2-AG** | 1,658 (p=0,202) | **3,700 (p<0,01)** | 0,153 (p=0,961) | 1/78 |
|  |  |  |  |  |  |
|  | **OEA** | **81,631 (p<0,001)** | **3,371 (p<0,05)** | 1,386 (p=0,248) | 1/79 |
| **HIPPO** | **AEA** | **8,068 (p<0,01)** | **2,922 (p<0,05)** | 2,241 (p=0,073) | 1/79 |
|  | **PEA** | 0,000 (p=0,999) | **3,482 (p<0,05)** | 0,129 (p=0,971) | 1/79 |
|  | **LEA** | 2,458 (p=0,121) | **4,854 (p<0,01)** | 0,135 (p=0,969) | 1/79 |
|  | **SEA** | 0,425 (p=0,517) | **5,227 (p<0,01)** | 0,496 (p=0,739) | 1/79 |
|  | **2-AG** | 3,539 (p=0,064) | 0,523 (p=0,719) | 0,936 (p=0,448) | 1/79 |

**Table S3 Two-way ANOVA results of NAEs and 2-AG in plasma and** **selected brain areas**

Results of the two-way ANOVA analyses of NAEs and 2-AG distribution, in plasma and in different brain areas of rats sacrificed at different time points after intraperitoneal injection of either OEA (10 mg kg^-1^) or vehicle VEH. Area postrema (AP), nucleus of the solitary tract (NST), arcuate nucleus/median eminence (Arc/ME), hippocampus (HIPPO), anandamide (AEA), palmitoylethanolamide (PEA), stearoyletanolamide (SEA), linoleoylethanolamide (LEA) and 2-arachidonoylglycerol (2-AG).

|  |  | **2.5 min** | | **5 min** | | **15 min** | | **30 min** | | **60 min** | |
| --- | --- | --- | --- | --- | --- | --- | --- | --- | --- | --- | --- |
|  |  | **VEH** | **OEA** | **VEH** | **OEA** | **VEH** | **OEA** | **VEH** | **OEA** | **VEH** | **OEA** |
| **PLASMA (pmol/ml)** | **OEA** | 16,53 ± 0,7829 | **550,8 ± 87,80***** | 16,99 ± 0,7457 | **735,4 ± 38,96***** | 15,85 ± 0,819 | **985,6 ± 67,17***** | 16,53 ± 1,283 | **1057 ± 56,82***** | 18,72 ± 0,7616 | **540,0 ± 70,25***** |
|  | **AEA** | 11,57 ± 0,6142 | 11,09 ± 0,5882 | 11,72 ± 0,4475 | 12,87 ± 0,4514 | 11,15 ± 0,8630 | **14,92 ± 1,029**** | 9,373 ± 0,5478 | **13,27 ± 1,348**** | 12,20 ± 0,5647 | 13,92 ± 1,005 |
|  | **PEA** | 54,68 ± 2,090 | 61,85 ± 2,573 | 54,49 ± 3,200 | **71,92 ± 3,110***** | 59,04 ± 5,137 | **82,33 ± 2,979***** | 55,22 ± 2,668 | **75,14 ± 3,340***** | 57,25 ± 1,959 | **68,76 ± 4,170*** |
|  | **LEA** | 6,699 ± 0,4407 | **49,47 ± 7,310***** | 6,361 ± 0,3202 | **71,31 ± 5,196***** | 5,945 ± 0,3635 | **72,98 ± 8,052***** | 6,025 ± 0,4193 | **55,34 ± 6,428***** | 6,641 ± 0,4429 | **20,83 ± 1,626*** |
|  | **SEA** | 6,361 ± 0,6311 | **10,95 ± 0,7400**** | 6,913 ± 0,9703 | **13,55 ± 1,287***** | 7,744 ± 1,338 | **20,77 ± 0,8551***** | 8,516 ± 1,164 | **31,64 ± 1,111***** | 7,174 ± 0,4828 | **28,38 ± 1,456***** |
|  | **2-AG** | 63,16 ± 0,3439 | 62,97 ± 0,5491 | 62,96 ± 0,3059 | 63,06 ± 0,3938 | 64,73 ± 0,6939 | **62,95 ± 0,3742**** | 63,06 ± 0,1916 | 63,69 ± 0,2118 | 62,40 ± 0,2186 | 62,83 ± 0,3731 |
| **AP (pmol/g)** | **OEA** | 102,1 ± 19,28 | 132,2 ± 17,57 | 84,32 ± 9,341 | **164,9 ± 19,43*** | 81,35 ± 16,51 | **248,4 ± 43,01***** | 198,5 ± 29,13 | 268,0 ± 46,11 | 104,2 ± 19,57 | 178,7 ± 27,12 |
|  | **AEA** | 123,3 ± 21,43 | 112,9 ± 25,12 | 104,7 ± 18,02 | 96,35 ± 13,43 | 115,4 ± 9,720 | 118,1 ± 26,27 | 118,1 ± 27,14 | 109,2 ± 19,12 | 91,86 ± 14,19 | 109,8 ± 16,98 |
|  | **PEA** | 1160 ± 208,3 | 1160 ± 328,7 | 1211 ± 250,5 | 1091 ± 171,1 | 1033 ± 93,74 | 1123 ± 281,2 | 1282 ± 232,0 | 1241 ± 295,9 | 961,6 ± 148,7 | 1123 ± 230,9 |
|  | **LEA** | 58,50 ± 10,80 | 50,00 ± 6,736 | 57,42 ± 9,195 | 60,03 ± 8,034 | 48,64 ± 4,832 | 53,44 ± 14,93 | 79,03 ± 15,15 | 76,26 ± 20,92 | 61,69 ± 10,60 | 63,67 ± 12,41 |
|  | **SEA** | 179,6 ± 41,28 | 152,9 ± 54,43 | 164,6 ± 44,80 | 163,7 ± 34,32 | 174,8 ± 19,22 | 163,4 ± 29,65 | 171,4 ± 42,56 | 189,3 ± 51,73 | 114,9 ± 17,87 | 154,9 ± 36,66 |
|  | **2-AG** | 4120 ± 460,0 | 3696 ± 516,6 | 3767 ± 307,3 | 4083 ± 294,9 | 4261 ± 459,0 | 3601 ± 547,3 | 3343 ± 373,1 | 3122 ± 307,2 | 2944 ± 309,7 | 3559 ± 453,6 |
| **NST (pmol/g)** | **OEA** | 553,2 ± 83,29 | 512,2 ± 26,41 | 454,4 ± 13,54 | **635,6 ± 47,30**** | 460,2 ± 12,54 | **766,6 ± 25,55***** | 562,8 ± 74,80 | **834,8 ± 39,94***** | 466,9 ± 11,30 | **651,4 ± 37,44**** |
|  | **AEA** | 122,7 ± 40,77 | 88,08 ± 15,44 | 117,1 ± 24,59 | 92,34 ± 19,88 | 123,4 ± 10,51 | 113,7 ± 15,99 | 123,1 ± 16,57 | 116,9 ± 27,39 | 85,36 ± 6,837 | 77,11 ± 7,696 |
|  | **PEA** | 2612 ± 296,3 | 1721 ± 131,2 | 2035 ± 58,58 | 1982 ± 128,7 | 1997 ± 68,67 | 2032 ± 99,74 | 2413 ± 325,2 | 2560 ± 261,9 | 2156 ± 86,99 | 2156 ± 71,24 |
|  | **LEA** | 44,39 ± 14,38 | 59,24 ± 17,80 | 38,52 ± 10,70 | 50,23 ± 7,541 | 31,58 ± 3,873 | 38,47 ± 2,537 | 78,70 ± 18,75 | 69,42 ± 16,26 | 34,81 ± 9,316 | 30,15 ± 4,898 |
|  | **SEA** | 419,5 ± 44,69 | 300,0 ± 60,39 | 373,7 ± 53,00 | 380,8 ± 46,48 | 382,8 ± 15,27 | 435,4 ± 35,65 | 349,1 ± 67,55 | 407,7 ± 28,38 | 430,5 ± 16,44 | 441,2 ± 24,51 |
|  | **2-AG** | 15120 ± 2371 | 11909 ± 1528 | 14238 ± 2189 | 11503 ± 827,3 | 12524 ± 829,2 | 9473 ± 1066 | 13684 ± 654,1 | 11351 ± 620,3 | 9961 ± 233,2 | 9648 ± 554,8 |
| **Arc/ME (pmol/g)** | **OEA** | 230,8 ± 24,07 | **297,8 ± 16,01*** | 218,9 ± 8,964 | 274,1 ± 16,24 | 240,2 ± 22,25 | **377,4 ± 15,74***** | 238,2 ± 27,03 | **365,6 ± 24,32***** | 181,5 ± 8,532 | **300,4 ± 24,00***** |
|  | **AEA** | 77,30 ± 7,907 | 62,59 ± 7,913 | 81,02 ± 5,052 | 81,75 ± 13,88 | 80,71 ± 10,67 | 74,48 ± 9,919 | 89,92 ± 8,631 | 80,55 ± 8,880 | 60,68 ± 3,951 | 66,65 ± 6,652 |
|  | **PEA** | 739,3 ± 86,20 | 718,7 ± 38,79 | 789,7 ± 73,49 | 601,6 ± 29,28 | 690,8 ± 72,18 | 756,6 ± 65,28 | 709,5 ± 99,02 | 572,3 ± 41,25 | 618,6 ± 48,45 | 659,1 ± 70,84 |
|  | **LEA** | 22,47 ± 4,841 | 24,04 ± 5,469 | 12,81 ± 2,007 | 22,34 ± 3,568 | 19,75 ± 4,666 | 20,91 ± 1,750 | 15,02 ± 2,957 | 25,63 ± 4,123 | 18,31 ± 3,417 | 21,16 ± 9,584 |
|  | **SEA** | 101,5 ± 13,00 | 94,69 ± 11,79 | 116,6 ± 15,03 | 78,30 ± 10,44 | 78,79 ± 15,70 | 112,5 ± 22,56 | 154,1 ± 16,57 | 153,4 ± 9,825 | 138,0 ± 12,63 | 149,5 ± 9,303 |
|  | **2-AG** | 26660 ± 4070 | 26174 ± 2593 | 26590 ± 2515 | 24821 ± 2651 | 26337 ± 2656 | 22564 ± 1609 | 29630 ± 3351 | 26045 ± 2926 | 19163 ± 2044 | 18018 ± 1309 |
| **HIPPO (pmol/g)** | **OEA** | 147,2 ± 6,362 | **202,7 ± 12,59**** | 159,9 ± 13,01 | **224,4 ± 14,65**** | 163,4 ± 5,877 | **272,1 ± 18,45***** | 168,3 ± 8,584 | **251,6 ± 15,22***** | 176,3 ± 10,10 | **237,4 ± 18,42**** |
|  | **AEA** | 142,5 ± 10,36 | 156,8 ± 10,31 | 163,5 ± 8,616 | 136,6 ± 4,314 | 158,7 ± 15,27 | 132,6 ± 9,416 | 170,7 ± 13,17 | **126,2 ± 6,099**** | 125,8 ± 14,37 | 115,4 ± 6,141 |
|  | **PEA** | 593,0 ± 42,13 | 615,5 ± 44,45 | 601,0 ± 54,11 | 586,5 ± 43,73 | 641,8 ± 24,35 | 665,9 ± 74,18 | 685,4 ± 68,60 | 647,6 ± 42,04 | 762,1 ± 40,17 | 768,1 ± 62,32 |
|  | **LEA** | 11,54 ± 0,6578 | 13,13 ± 0,4788 | 12,17 ± 0,8385 | 14,94 ± 1,328 | 13,54 ± 1,315 | 15,02 ± 0,7360 | 18,06 ± 2,498 | 19,57 ± 2,575 | 14,37 ± 1,575 | 14,83 ± 1,997 |
|  | **SEA** | 80,51 ± 9,880 | 91,00 ± 8,295 | 83,87 ± 9,594 | 78,39 ± 9,017 | 88,63 ± 6,813 | 94,62 ± 12,50 | 111,1 ± 19,47 | 104,0 ± 6,922 | 114,7 ± 8,453 | 133,4 ± 12,26 |
|  | **2-AG** | 8617 ± 258,4 | 7569 ± 313,6 | 7957 ± 434,9 | 8414 ± 687,1 | 8211 ± 548,9 | 7170 ± 299,6 | 8506 ± 571,3 | 8119 ± 472,5 | 8418 ± 414,3 | 7729 ± 363,3 |

**Table S4 Concentration of NAEs and 2-AG in plasma and** **selected brain areas after intraperitoneal injection of OEA or VEH.**

Concentration values of NAEs and 2-AG in plasma (pmol/ml) and brain areas (pmol/g) after intraperitoneal injection of OEA or VEH. Data are expressed as mean ± SEM. * p<0,05, ** p<0,01, *** p<0,001 vs VEH in the same time point (Tukey’s test). Area postrema (AP), nucleus of the solitary tract (NST), arcuate nucleus/median eminence (Arc/ME), hippocampus (HIPPO), anandamide (AEA), palmitoylethanolamide (PEA), stearoyletanolamide (SEA), linoleoylethanolamide (LEA) and 2-arachidonoylglycerol (2-AG).
